# Arrangements of proteins at reconstituted synaptic vesicle fusion sites depend on membrane separation

**DOI:** 10.1002/1873-3468.13916

**Published:** 2020-09-12

**Authors:** Lucy Ginger, Joerg Malsam, Andreas F.‐P. Sonnen, Dustin Morado, Andrea Scheutzow, Thomas H. Söllner, John A. G. Briggs

**Affiliations:** ^1^ MRC Laboratory of Molecular Biology Cambridge UK; ^2^ Heidelberg University Biochemistry Center Heidelberg Germany; ^3^ Structural and Computational Biology Unit European Molecular Biology Laboratory Heidelberg Germany; ^4^ Present address: Department of Pathology University Medical Centre Utrecht Utrecht University Utrecht 3508 GA the Netherlands

**Keywords:** cryoelectron tomography, fusion, *in vitro* reconstitution, membrane, SNARE, synaptic vesicle

## Abstract

Synaptic vesicle proteins, including N‐ethylmaleimide‐sensitive factor attachment protein receptors (SNAREs), Synaptotagmin‐1 and Complexin, are responsible for controlling the synchronised fusion of synaptic vesicles with the presynaptic plasma membrane in response to elevated cytosolic calcium levels. A range of structures of SNAREs and their regulatory proteins have been elucidated, but the exact organisation of these proteins at synaptic junction membranes remains elusive. Here, we have used cryoelectron tomography to investigate the arrangement of synaptic proteins in an *in vitro* reconstituted fusion system. We found that the separation between vesicle and target membranes strongly correlates with the organisation of protein complexes at junctions. At larger membrane separations, protein complexes assume a ‘clustered’ distribution at the docking site, inducing a protrusion in the target membrane. As the membrane separation decreases, protein complexes become displaced radially outwards and assume a ‘ring‐like’ arrangement. Our findings indicate that docked vesicles can possess a wide range of protein complex numbers and be heterogeneous in their protein arrangements.

## Graphical abstract

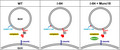

## Abbreviations

**Cpx**, Complexin

**CCs**, correlation coefficients

**cryo‐EM**, cryo‐electron microscopy

**cryo‐ET**, cryo‐electron tomography

**GUVs**, giant unilamellar vesicles

**PIP_2_**, phosphatidylinositol‐4,5‐bisphosphate

**SNAREs**, N‐ethylmaleimide‐sensitive factor attachment protein receptors

**SUVs**, small unilamellar vesicles

**Syt1**, Synaptotagmin 1

**WT**, wild‐type

Vesicle fusion occurs when the membrane of a vesicle merges with the membrane of an organelle or plasma membrane. In doing so, fusion allows the contents of vesicles and lipids to be transferred between intracellular and extracellular compartments [1]. In neurons, docked and primed synaptic vesicles are ready to fuse with the presynaptic membrane. Fusion occurs rapidly and in a synchronised manner in response to locally elevated calcium levels, releasing neurotransmitters into the synaptic cleft [2]. Fusion is enabled by N‐ethylmaleimide‐sensitive factor attachment protein receptors (SNAREs). T‐SNAREs composed of syntaxin 1A and SNAP‐25 on the plasma membrane interact and fold with the v‐SNARE VAMP2/synaptobrevin on synaptic vesicles [3]. Folding occurs in a zipper‐like fashion beginning at the N termini of SNAREs and proceeding towards their C termini, reducing the distance between the apposing lipid bilayers and resulting in the formation of a four‐helical bundle trans‐SNARE complex (SNAREpin) [4, 5]. The cytoplasmic region of each SNARE protein contains a ‘SNARE’ motif consisting of 15 conserved hydrophobic layers (positions −7 to −1 and +1 to +8) and an ionic layer at position 0. Once folded, these layers form the largely hydrophobic core of the trans‐SNARE complex [6, 7]. The helical folding extends to the pretransmembrane linker and releases sufficient energy to overcome the energetic barrier separating the vesicle and plasma membrane, thereby allowing it to drive fusion [4, 8, 9, 10].

Numerous regulatory factors assist in promoting formation of the trans‐SNARE complex and later in precisely coordinating its response to calcium influx. Briefly, initial vesicle tethering is likely mediated by the interaction of Rab3 on synaptic vesicles with Rim1 at the active zone of the plasma membrane [11]. Subsequent priming reactions involving Munc13‐1 and Munc18‐1 control the N‐terminal assembly of the trans‐SNARE complex [12, 13, 14, 15, 16, 17, 18, 19, 20, 21]. Ca^2+^ regulation requires Synaptotagmin 1 (Syt1) and Complexin (Cpx) [22, 23, 24, 25, 26, 27]. Syt1 is an integral membrane protein present alongside VAMP2 in synaptic vesicles, and it possesses two cytoplasmic C2 domains that bind Ca^2+^ [9, 22]. In addition to Ca^2+^ binding, Syt1 uses its N‐terminal C2B domain to bind phosphatidylinositol‐4,5‐bisphosphate (PIP_2_) on the plasma membrane. Syt1‐PIP_2_ and Syt1‐t‐SNARE binding contribute to the docking of vesicles to plasma membranes [28, 29]. The small cytosolic protein Cpx helps to coordinate proper trans‐SNARE zippering through a series of interactions with the forming trans‐SNARE complex [30, 31, 32]. While Cpx has a prominent stimulatory function in Ca^2+^‐dependent neurotransmitter release, Cpx also acts to inhibit the complete folding of the trans‐SNARE through binding to the membrane proximal regions of SNAP‐25 and VAMP2 [33, 34]. Such binding blocks the C‐terminal folding of the trans‐SNARE complex, holding it in a partially folded clamped‐like state, preventing membrane fusion. Syt1 also contributes to the inhibitory clamp through interactions between its C2B domain, the trans‐SNARE and Cpx [35]. In this way, the synaptic vesicle is trapped in a docked and primed state on the plasma membrane [35]. Through a poorly understood mechanism, during Ca^2+^ influx the C2 domains of Syt1 bind Ca^2+^ and anionic lipids causing conformational changes that allow the complete folding/zippering of the trans‐SNARE complex to bring the two membranes into close proximity and subsequently drive their fusion [35, 36, 37, 38].

Despite the biochemistry of synaptic proteins being well understood, their organisation at fusion junctions has proven more difficult to decipher. Several structural studies have tried to address this. Crystal structures have revealed two possible Syt1 binding sites on either side of prefusion SNARE–Cpx complexes [39, 40] and cryoelectron microscopy (cryo‐EM) studies have shown that Syt1 oligomerises into rings when added to monolayers [41]. These observations led to the proposal of a ‘buttress ring’ model for synaptic protein organisation in which SNAREpins are bound to the plasma membranes via a Syt1 oligomeric ring, and are simultaneously flanked by Syt1 monomers on their vesicle‐facing side [35, 42, 43]. Furthermore, a recent cellular cryoelectron tomography (cryo‐ET) study suggested a sixfold symmetrical arrangement of synaptic protein complexes in this primed prefusion vesicle state [44].

Other insights into fusion protein organisation and function have been obtained using synaptic proteins reconstituted into liposomes or other membranes. These systems can be used for quantitative studies of fusion kinetics as well as for structural studies by cryo‐ET. For example, one study suggested that smaller ‘point’ contacts representing lower numbers of protein complexes are preferential for membrane fusion and that higher order assemblies of complexes are delirious to fusion [45]. On the other hand, a separate cryo‐EM study indicated that the partial folding of trans‐SNAREs forces opposing membranes tightly together prior to fusion [46]. Both of these studies were limited in that both t‐SNARE and v‐SNARE proteins were reconstituted into small unilamellar vesicles (SUVs). SUVs reconstituted with the t‐SNARE exhibit high membrane curvatures that do not accurately mimic the relatively flat surface of the plasma membrane. This higher membrane curvature may limit the ability of fusion junction proteins to properly organise. Here, we use cryo‐ET to address fusion protein organisation by analysing an *in vitro* reconstituted system [47] where VAMP2 and Syt1 were reconstituted into SUVs, while preformed t‐SNARE complexes of syntaxin 1A and SNAP‐25 were reconstituted into giant unilamellar vesicles (GUVs). The large size and lower membrane curvature of the GUVs were designed to mimic the relatively flat surface of the plasma membrane, while SUVs ranging from roughly 20–150 nm diameter mimicked synaptic vesicles. This system has been previously used in cryo‐ET studies to investigate membrane morphologies at fusion junctions [48]. Here, taking advantage of improved cryo‐EM methods, we were able to analyse the distribution of protein densities at vesicle docking sites in more detail and distinguish three types of protein density organisation classes at vesicle docking sites. We found that the membrane morphology, protein arrangement and the distance between SUV and GUV membranes are intimately linked. Furthermore, we found that fusion sites have highly heterogeneous protein arrangements and numbers.

## Materials and methods

### Protein reconstitution and fusion assays

**Fig. 1:**
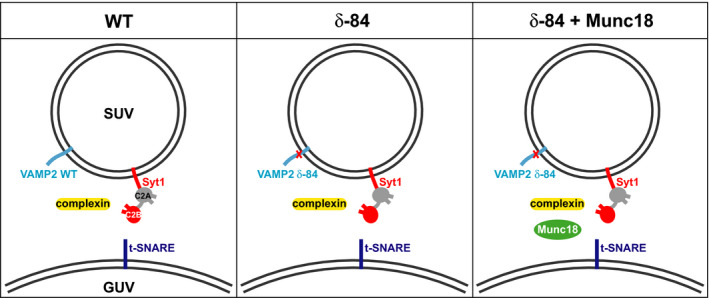
Schematic representations of the three sample conditions. The components of the WT, δ‐84 and δ‐84 + Munc18 conditions are shown. VAMP2/VAMP2 δ‐84 (light blue) and Syt1 (red, with C2A domain shown as grey) were reconstituted into SUVs. The location of the L84 deletion mutation of VAMP2 δ‐84 is shown (red cross). GUVs were reconstituted with t‐SNARE (dark blue). Complexin was added to the solutions of all three conditions (yellow). In the case of the δ‐84 + Munc18 condition, Munc18 (green) was added to the solution.

In this study, we used the major protein isoforms involved in synaptic vesicle fusion. Among the two Cpx isoforms involved in neurotransmitter release, we chose CpxII [49]. Wild‐type VAMP2, syntaxin 1A, SNAP‐25, CpxII and Syt1 were expressed and purified using methods described previously [47]. A single amino acid deletion mutant in the +8 layer of VAMP2 (VAMP2 δ‐84) was also expressed and purified using the same methods used for wild‐type VAMP2. Munc‐18 was expressed and purified according to [50]. Wild‐type VAMP2 or the VAMP2 δ‐84 mutant were reconstituted together with Syt1 into SUVs, while preformed t‐SNARE complexes of syntaxin 1A and SNAP‐25 were reconstituted into GUVs following methods described previously [47]. GUVs (250 nm t‐SNARE, 250 µm lipid) were incubated with SUVs (70 nm Syt1, 140 nm VAMP2, 50 µm lipid) and the regulatory protein CpxII (6 µm) for 5 min on ice to allow docking. These concentrations of lipids and proteins correspond to ~ 44 outward facing v‐SNAREs and 22 Syt1 molecules in an 80 nm diameter SUV, and ~ 15 outward facing t‐SNAREs in an equivalent area of GUV membrane. Subsequently, fusion competency was assessed using fluorescence dequenching lipid mixing fusion assays as described previously [47]. Where indicated, the regulator Munc‐18 was also mixed into GUV solutions at 0.75 µm concentration. Samples were prepared under three different conditions: ‘WT’ consisting of VAMP2/Syt1 SUVs + t‐SNARE GUVS + CpxII, ‘δ‐84’ consisting of δ‐84 VAMP2/Syt1 SUVs + t‐SNARE GUVs + CpxII, and ‘δ‐84 + Munc18’ consisting of δ‐84 VAMP2/Syt1 SUVs + t‐SNARE GUVs + CpxII + Munc18‐1 Fig. 1.

### Cryo‐EM sample preparation

SUV/GUV mixtures were incubated on ice, and the WT and δ‐84 samples were incubated for 15 min, while the δ‐84 + Munc18 sample was incubated for 1 h. Following incubation on ice, samples underwent a 1 min 37 °C warm‐up. Ten nanometre gold fiducials were then added to samples. Three microlitre of each sample was applied to glow‐discharged lacey 200 mesh carbon grids (Plano GmbH, Wetzlar, Germany) before being manually back side blotted in a high humidity chamber (EMBL, Heidelberg, Germany) using Whatman 1 filter paper that had been previously soaked in 50 mm EDTA and then dried. Following blotting, grids were plunged and frozen in liquid ethane.

### Cryo‐ET data acquisition

Tomograms were collected on a Titan Krios electron microscope (Thermo Fisher, Waltham, MA, USA) operated at 300 kV using a Volta phase plate and a K2 Summit direct electron detector (Gatan, Pleasanton, CA, USA). Tilt series images were acquired using SerialEM software [51] at 0 μm defocus, 81 000× magnification, 70 μm objective aperture, 50 μm C2 aperture, energy filter slit width of 20 eV and pixel sizes ranging from 4.35 to 5.31 Å/pixel. Dose‐symmetric tilt series [52] with a 1° step were collected over +/− 60 degrees. The total electron dose was ~ 200 e^‐^/Å^2^. See Table 1 for a summary of data collection parameters.

**Table 1:** Cryo‐ET data collection parameters.

Condition	WT	δ‐84	δ‐84	δ‐84 + Munc‐18
Voltage (kV)	300	300	300	300
Volta phase plate	Yes	Yes	Yes	Yes
Detector	Gatan K2	Gatan K2	Gatan K2	Gatan K2
Energy filter slit width (eV)	20	20	20	20
Electron exposure (e/Å^2^)	~ 200	~ 200	~ 200	~ 200
Defocus (μm)	0	0	0	0
Tilt range (min/max, step)	−60°/+60°, 1°	−60°/+60°, 1°	−60°/+60°, 1°	−60°/+60°, 1°
Tilt scheme	Dose‐symmetrical (Hagen Scheme)	Dose‐symmetrical (Hagen Scheme)	Dose‐symmetrical (Hagen Scheme)	Dose‐symmetrical (Hagen Scheme)
Tomogram used/acquired	18/21	8/15	5/5	17/21
Pixel size (Å)	5.31	4.51	4.80	4.35
Docked vesicles analysed	155	64	26	133

### Image processing

Tilt series were aligned and reconstructed using TomoAlign [53] and IMOD 4.10.30 [54]. Tomograms were reconstructed with fourfold binning by Weighted Back Projection (WBP) implementing a SIRT emulating filter (20 iterations). Tomograms were visualised in Amira 6.0 (Thermo Fisher), and a 3 × 3 × 3 3D Gaussian filter was applied.

### Image analysis

Owing to a large difference in size, most GUVs and SUVs were easily distinguishable. Docking sites were defined as those where SUVs were found within 50 nm of GUVs, and where visible protein density was found between the SUV and GUV membranes. A small number of sites were excluded from further analysis because there was no obvious size difference between contacting vesicles or because overlapping features prevented clear interpretation. At SUV‐GUV docking sites, landmark points were placed at the centres of SUVs and at a position midway between SUV and GUV membranes at their point of closest approach. Individual subtomograms of junctions were extracted as 150 nm boxes, centred on the second landmark point. The subtomograms were rotated to place the vector between the two landmark points on the z‐axis. Additional landmark points were then placed at the centres of all protein densities observed on the SUV membrane, GUV membrane and in the intermembrane space. These landmark points were used to quantify the number of protein densities. Due to the limited signal‐to‐noise ratio in cryoelectron tomograms, and the application of image filters, observed density may correspond to a single protein or to more than one protein if they are too close together to be separately resolved. Small or extended proteins may not give rise to a visible density. The number of protein densities is therefore lower than the number of proteins. The number of protein densities will, however, correlate with the number of proteins, thereby allowing comparison between states and stages can be made. A landmark point was added on the SUVs outer membrane leaflet, at the point of closest approach to the GUV membrane, and the distance from this point to the GUV membrane was then measured using the Amira distance measuring tool.

Junction morphologies were visually assessed and categorised as either ‘Clustered’ (0), ‘Intermediate’ (0.5) or ‘Ring‐like’ (1) based on the distribution of landmark protein density points at junctions. The presence or absence of GUV membrane protrusions at each junction was visually assessed and categorised as (1) and (0), respectively. Pearson correlation coefficients (CCs) were then calculated between the measured parameters: junction morphology, distance between GUV‐SUV outer membrane leaflets, GUV membrane protrusions and the total number of protein densities at junctions. Regression analysis was performed to test the significance of any observed correlations. We classified significant CCs with magnitudes between 0.1 and 0.3 as ‘small’, between 0.3 and 0.5 as ‘moderate’ and above 0.5 as ‘strong’ [55].

### Averaging of junction morphology classes

To assess relationships between junction morphology, membrane separation and GUV protrusions, subtomograms of each junction type from the WT condition were brought into alignment using previously added landmark points placed on SUV bases, and subtomogram volumes were averaged using MATLAB. The coordinate points of protein densities from all subtomograms of each junction type were overlaid onto the averaged SUV volume densities and visualised in Chimera.

### The data set as a resource

The tomograms we have generated can act as a data resource against which other models can be compared, or from which models can be derived. We have therefore deposited the reconstructed tomograms, as well as the landmark points which mark the position and orientations or docking sites, at the EMPIAR database (accession number EMPIAR‐10498).

## Results

### Morphology of docked, primed vesicles in a reconstituted system

CpxII was mixed with GUVs containing syntaxin 1A and SNAP‐25 (reconstituted as a preassembled complex) in calcium‐free solutions. SUVs containing Syt1 and VAMP2 were mixed with CpxII‐GUV solutions and incubated on ice before undergoing a 1 min 37 °C warm‐up. In an attempt to improve sample homogeneity, and therefore interpretability, by generating more uniformly zippered trans‐SNARE complexes, in some samples VAMP2 was replaced with a fusion‐incompetent deletion mutant, VAMP2 δ‐84. VAMP2 δ‐84 lacks L84 at the +8 position of the SNARE motif and locks trans‐SNARE zippering near the membrane proximal C‐terminal end of the SNARE motifs [46]. In addition, Munc18‐1 was added to one reaction mix to increase the total protein density at docking sites in an attempt to further improve interpretability – this condition was incubated for an extended time period of 1 h to increase binding of Munc18‐1 to the SNARE complex. Samples were plunge‐frozen for cryo‐EM under three different conditions. The conditions are referred to as ‘WT’ (VAMP2/Syt1 SUVs + t‐SNARE GUVS + CpxII, incubated for 15 min), ‘δ‐84’ (VAMP2 δ‐84/Syt1 SUVs + t‐SNARE GUVs + CpxII, incubated for 15 min); and ‘δ‐84 + Munc18’ (VAMP2 δ‐84/Syt1 SUVs + t‐SNARE GUVs + CpxII + Munc18‐1) Fig. 1. Samples were imaged by cryo‐ET using a Volta phase plate. Tomograms were aligned and reconstructed to generate 3D representations of the docked, primed vesicles, see Materials and methods.

**Fig. 2:**
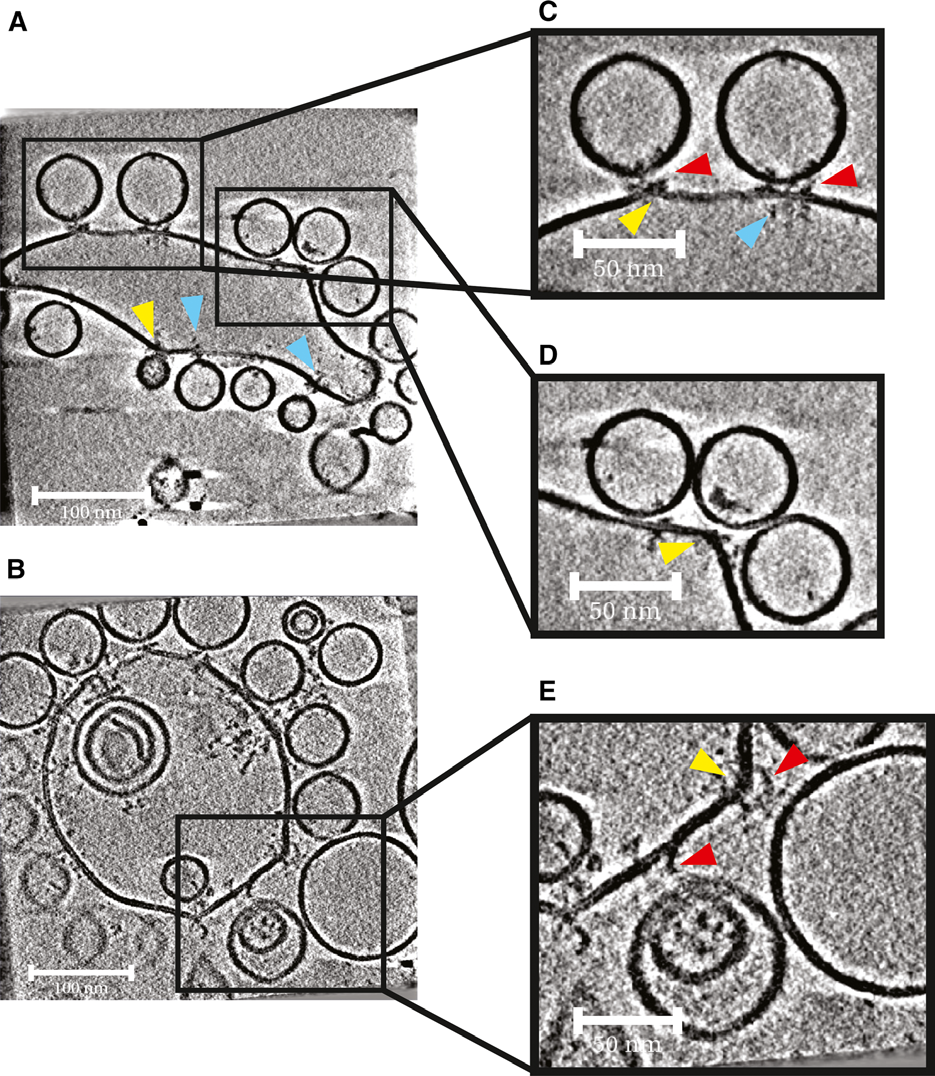
Morphology of vesicle docking sites. Slices from tomograms of sites where SUVs were observed docked to GUVs. Some GUVs appeared more tubulated (A), while other GUVs were more spherical in appearance (B). Protein complexes were observed on the inner leaflets of GUVs clustered around SUV‐GUV docking sites (blue arrowheads). (C) Protein complexes were observed on outer leaflets of GUVs, clustered around sites where SUVs were docked to GUVs (red arrowheads). (D) At sites where SUVs were docked, GUV membrane protrusions were sometimes observed (yellow arrowheads). (E) GUV membrane protrusions were often seen at sites where an SUV was docked at a large distance from the GUV. Scale bars in A and B = 100 nm; scale bars in C, D and E = 50 nm.

Giant unilamellar vesicle morphologies are variable, and some GUVs appear completely spherical, while others are tubulated or have irregular curvatures Fig. 2A,B. SUVs were spherical and on average 80 nm in diameter but had a size range of 20–150 nm.

A small number of t‐SNARE complexes could be seen protruding from the external and the internal surfaces of GUVs at sites where no docked SUVs were present. V‐SNARE and/or Syt1 were sometimes seen protruding from the internal and external surfaces of undocked SUVs. Protein densities at sites of docking were clearly seen both on SUV and GUV membranes and on some occasions in the space between SUV and GUV membranes Fig. 2A,B. Some docked SUVs appeared to be at larger distances from GUV membranes, while others appeared to be physically touching the GUV membrane. Where SUVs appeared docked, GUV membrane protrusions were sometimes seen (Fig. 2, yellow arrowheads). At larger membrane separations, there appeared to be a greater occurrence of GUV membrane protrusions. There were no obvious morphological differences between the three conditions.

At docking sites, t‐SNARE complexes appeared to cluster not only on the outer membrane leaflet, but also on the GUV inner membrane leaflet Fig. 2C, blue arrowheads). The inner membrane t‐SNARE clustering is independent of the presence of membrane protrusions or regions of higher membrane curvature. We propose that it results from antiparallel interactions between the transmembrane domains of t‐SNAREs that face outwards from the membrane, and t‐SNAREs that are incorporated into the membrane with the opposite orientation (facing inwards from the membrane) [56].

**Fig. 3:**
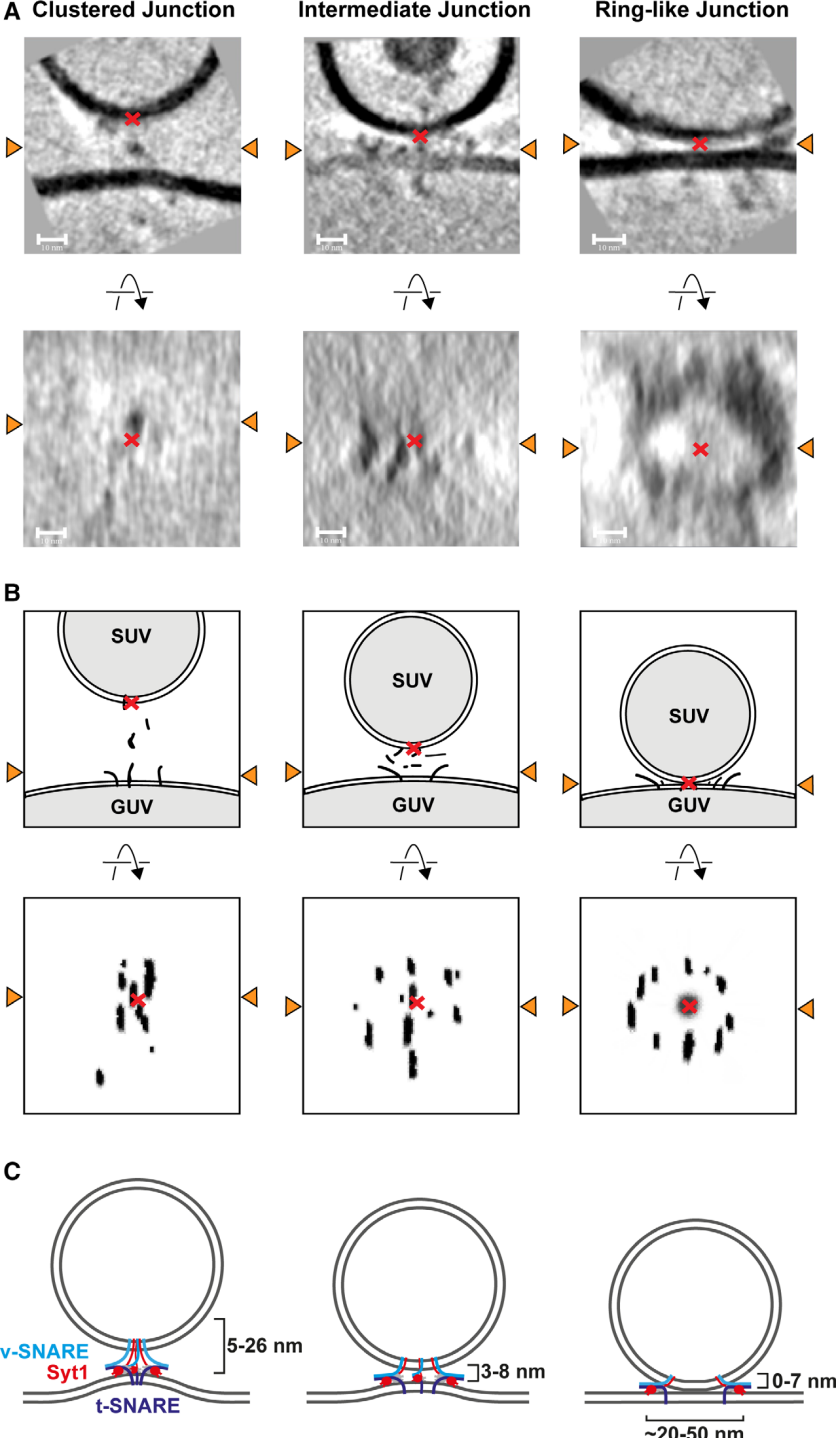
Examples of the three observed classes of junction and schematic representations thereof. (A) Slices from extracted subtomograms perpendicular to the membrane (upper row) and parallel to the membrane (lower row) illustrating the three junction morphologies observed across conditions. Orange arrowheads indicate the line at which the two slices intersect. Red crosses in the upper panels denote the base of SUVs, in lower panels crosses denote the position of the SUV base projected onto the slices. Electron densities in the lower panel of images appeared smeared in the z‐dimension owing to the missing wedge effect. Protein densities could be seen attached to GUV and SUV outer membranes, and between SUV and GUV membranes. Protein electron densities between the membranes may be anchored to membranes outside of the slice shown. Some densities do not appear to be anchored to membranes, suggesting they are connected via disordered protein chain which are not visible in the tomograms. Scale bars = 10 nm. Video S1 contains a 3D visualization of these volumes. (B) Cartoon representation of A. (C) Schematics of the three classes of junction. At membrane separations between 5 and 26 nm, clustered junctions are seen and local protrusions in the GUV membrane are formed. Intermediate junctions form at membrane separations between 3 and 8 nm. At membrane separations between 0 and 7 nm, ring‐like junctions are seen and GUV membrane protrusions are absent (v‐SNAREs light blue, t‐SNAREs dark blue, Syt1 red).

To facilitate further analysis of docking sites, we extracted and oriented docked SUV sites as subtomograms and inspected them using Amira (Materials and methods). We assessed the distribution of protein densities in relation to the point of closest approach between the SUV and GUV membranes. We observed three types of protein density organisation: ‘clustered junctions’ where protein densities were clustered at the point of closest approach between the GUV and SUV membranes and showed little organisation; ‘ring‐like junctions’ where all protein densities have been excluded from between the SUV‐GUV membranes and are arranged in a ring‐like fashion about the base of the SUV, leaving a region directly beneath the base of the SUV where no protein densities are observed; and ‘intermediate junctions’ where some but not all densities appeared to have become excluded from between the SUV and GUV membranes and have moved radially outward, while some densities are still observed beneath the SUV base Fig. 3 and Video S1. There appeared to be a correlation between membrane separation and the type of protein organisation seen: larger membrane separations correspond with clustered junctions, while junctions with smaller membrane separations seemed to be intermediate or ring‐like junctions. This was observed across all conditions. All junctions where SUV and GUV membranes directly contacted one another were ring‐like. Overall, no obvious morphological differences were observed between the three conditions.

### Quantitative characterisation of docking sites

**Fig. 4:**
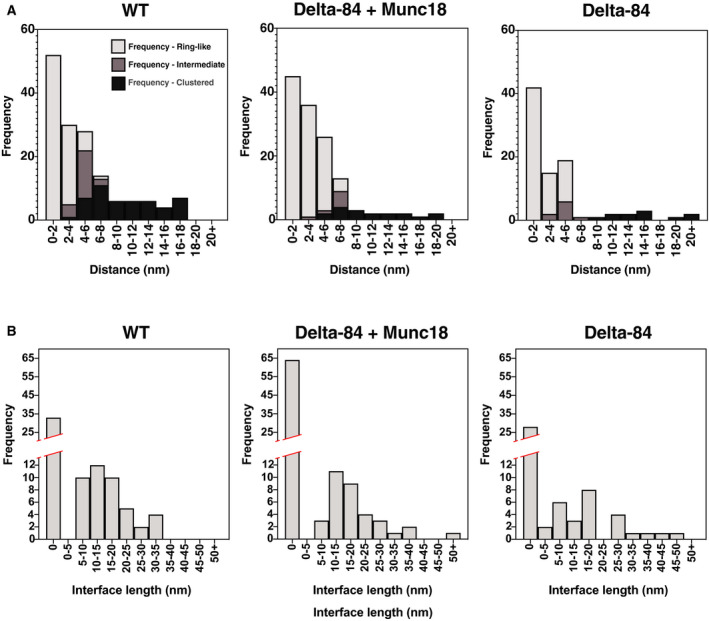
Measurements of membrane separations and membrane contact interfaces for different junctions. (A) Frequency of ring‐like, intermediate and clustered junctions found at given SUV‐GUV membrane separations. Stacked histograms are shown illustrating the distribution of membrane separation for the WT, δ‐84 + Munc18 and δ‐84 conditions. (B) Frequency of ring‐like junction interface diameters. Histograms are shown illustrating the distribution of diameters of the SUV‐GUV membrane–membrane contact region for ring‐like junctions in the WT, δ‐84 and δ‐84 + Munc18 conditions.

Our visual inspection of junctions suggested that there is a relationship between the distribution of protein densities and the separation between GUV and SUV membranes. To characterise this further, we measured the separation between outer leaflets of GUV and SUV membranes Fig. 4A (Materials and methods). At the majority of docking sites across all conditions, the membranes are separated by less than 10 nm (WT: 130 from 153, 85%, δ‐84: 78 from 88, 89%; δ‐84 + Munc‐18: 123 from 133, 92%) and only two junctions were identified with separations greater than 20 nm. Half‐zippered SNAREpins have been estimated to bridge a membrane separation of ~ 10 nm [57] – our data therefore suggest that most SNAREpins are at least zippered to the central 0‐layer of the SNARE motif.

We next analysed further the link between membrane separation and protein density distribution. We found clustered junctions at membrane separations of 5–26 nm, intermediate junctions at membrane separations of 3–8 nm and ring‐like junctions at membrane separations of 0–7 nm. In over 40% of ring‐like junctions, the GUV and SUV membranes were in direct contact, and in many cases, the contact involved large areas of membrane (contact diameters: WT 16 ± 8 nm, *n* = 51; δ‐84 = 18 ± 12 nm, *n* = 41; δ‐84 + Munc‐18 = 18 ± 10 nm, *n* = 34) Fig. 4B.

**Fig. 5:**
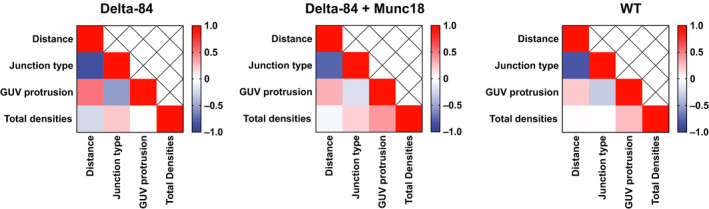
Heat maps depicting Pearson CCs between parameters measured from the WT, δ‐84 and δ‐84 + Munc18 conditions. Positive correlations are shown in red, negative correlations are shown in blue, and zero correlation is shown as white.

The distribution of membrane separations for different junction types was fairly consistent across all conditions Fig. 4A. Membrane separations > 8 nm, which are clustered junctions, were slightly more frequent in the WT condition than in the δ‐84 conditions Fig. 4A but this may reflect experimental variation. To quantitatively assess the relationship between junction morphology and membrane separation, Pearson CCs were calculated across all conditions. CCs were strong (between −0.76 and −0.86) and statistically significant (*t*‐test *P* values < 0.05) confirming the link between junction type and membrane separation Fig. 5.

**Fig. 6:**
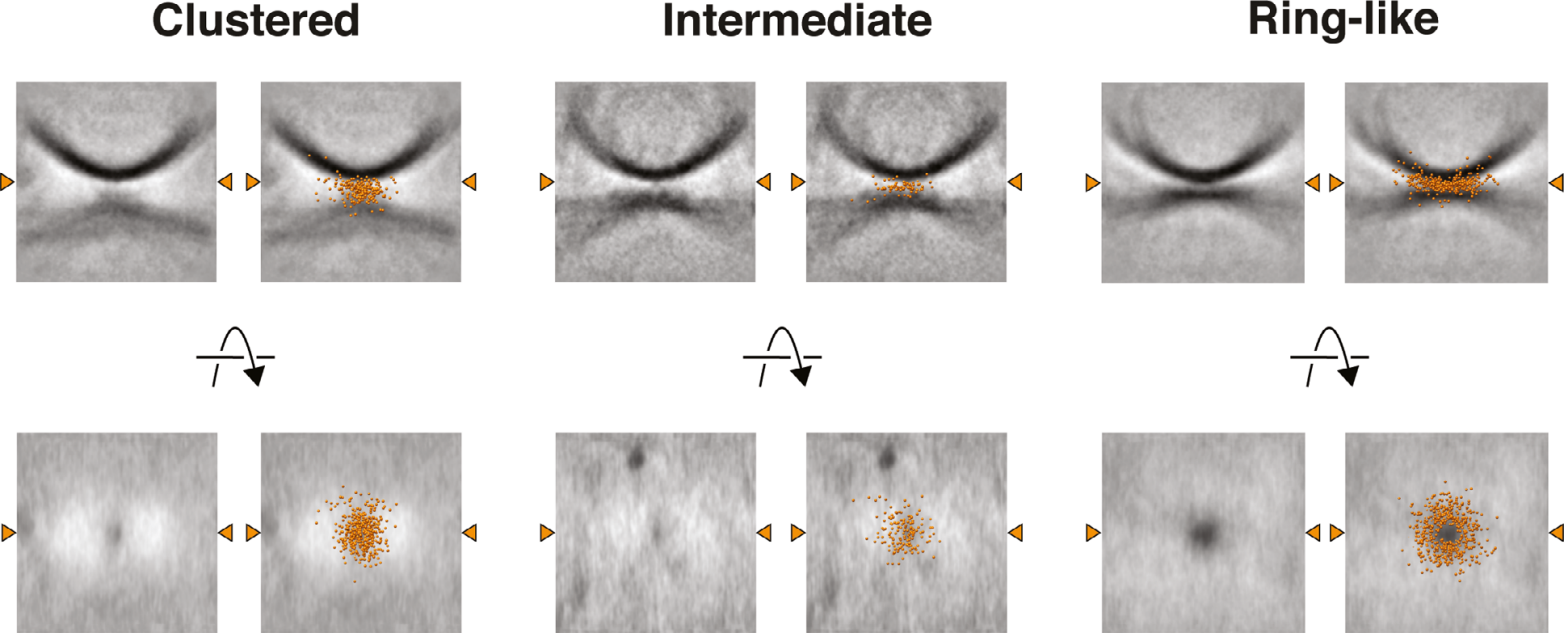
Averaged junction volumes from the ‘Clustered’, ‘Intermediate’ and ‘Ring‐like’ junctions. Individual subtomograms of each protein distribution junction class from the WT condition were aligned and averaged. In the right‐hand panels, the coordinate points of protein densities from all junctions of that class are overlaid onto the averaged volumes as orange spheres. Orange arrowheads indicate the line at which the two slices intersect. Video S2 contains a 3D visualisation of these volumes.

To better understand the protein distributions and membrane separations at different junction types, we aligned and averaged all subtomograms from the WT condition belonging to each junction type (Materials and methods). We additionally marked the coordinates of clearly visible protein densities within each subtomogram and displayed all coordinates from the subvolumes in each average Fig. 6, Video S2. The resulting averages reveal that from clustered to ring‐like junctions, there is a decrease in membrane separation and a redistribution of protein densities outwards away from the contact. The averaged clustered structure shows a protrusion in the GUV membrane that is absent in the averages of other junction types.

We considered the possibility that blotting of the sample during EM sample preparation might generate forces that pull tethered SUVs away from the GUV surface, and in so doing generate GUV protrusions. However, we observed that sites with protrusions can be adjacent to sites without protrusions although both should experience similar forces Fig. 2E. We found Pearson CCs between the presence of a GUV membrane protrusion and membrane separation of between 0.22 and 0.52, and between the presence of a GUV membrane protrusion and junction type of between −0.20 and −0.54 Fig. 5. There are therefore correlations between the presence of a GUV membrane protrusion, increasing membrane separation and junction type.

Ring‐like synaptotagmin oligomers have been proposed to play a role in regulating priming and fusion [58]. We therefore further analysed the protein distributions in ring‐like junctions. Ring‐like junctions are very heterogeneous in their structure and show different degrees of ‘completeness’. Some junctions showed almost complete rings of continuous protein density, while others showed only a small number of discrete protein densities spread out around the junction. Protein densities were found at different distances away from the point at which the base of the SUV was closest to the GUV. The distances of protein densities differed even within a docking site, meaning that in many cases, complete or partial protein rings were not perfectly circular Fig. S1. There was no obvious difference in the appearances of ring‐like junctions across the three conditions. When the SUV and GUV membranes were separated by < 3 nm, we only observed ring‐like junctions, while at membrane separations between 3 and 8 nm, we also observed intermediate junctions. Based on structural models, SNAREpin‐CpxII complexes have a diameter of ~ 2.5–3 nm across [59] (PDB 3rk3), while SNAREpin‐CpxII‐Syt1‐Syt1 complexes have a diameter of ~ 8 nm when measured across each SNAREpin‐flanking Syt1 C2B domain [39] (PDB 5cci). At membrane separations less than 8 nm, larger SNAREpin‐CpxII‐Syt1‐Syt1 complexes would therefore be excluded from the intermembrane space, while at separations < 3 nm, smaller complexes such as SNAREpin‐CpxII would also be excluded. Based on these observations, we propose that protein densities are excluded from between the SUV and GUV membranes when their membranes are brought close enough together, that this exclusion is largely determined by steric effects, and that exclusion generates the heterogeneous ring‐like morphologies we observe. The site‐specific arrest of SNAREpin zippering by the VAMP2 + 8 layer deletion mutant did not improve this heterogeneous morphology. Nevertheless, the addition of Ca^2+^ to this kind of reconstituted system causes nearly all docked SUVs containing wild‐type VAMP2 and Syt1 to fuse with t‐SNARE containing GUVs [47]. Thus, it is likely that the heterogeneous range of different ring‐like arrangements of protein densities that we observed are all capable of supporting vesicle fusion.

### Analysis of protein complex numbers at fusion sites

For each junction morphology, we analysed the number of electron densities that we attributed to protein complexes. Protein electron densities were assigned by identifying electron densities that were protruding from the surfaces of SUV and GUV membranes, or that were present in the space between GUV and SUV membranes. It should be noted that the precise number of protein densities counted will depend on the filtering applied to tomograms, and does not directly correspond to the number of proteins; however, the number of protein densities can be compared between docking sites (Materials and methods). The number of protein densities at clustered, intermediate and ring‐like junctions is highly variable, ranging from 2 to 34 protein densities per site Fig. S2. These observations suggest that docking and bringing the SUV and GUV membranes into very close proximity can be achieved using highly variable numbers of proteins.

The δ‐84 + Munc18 condition had a higher number of total protein densities compared to other conditions. The mean number of protein densities for the δ‐84 + Munc18 condition was 14.6 ± 6.9, *n* = 133, while for the δ‐84 condition, the mean was 10.1 ± 5.5, *n* = 90, and for the WT condition, the mean was 9.6 ± 4.4, *n* = 155. The larger number of protein densities observed presumably reflects the addition of Munc‐18 contributing densities to the docking site.

## Discussion

The organisation of protein complexes at fusion junctions in the primed prefusion state has remained controversial. Here, we have used a reconstituted system designed to mimic synaptic vesicle fusion that allows vesicles to be captured in their primed prefusion state. The membrane separations we observed are in the range of those seen in cryo‐ET studies of vitrified synapses, which found synaptic vesicles tethered to plasma membranes at distances ranging from 0 to 40 nm [60]. We observed that where vesicles are docked at larger distances from the target membrane, protein complexes cluster locally between the two membranes, and a protrusion is often seen in the target membrane. It has been suggested that formation of a local membrane protrusion may reduce the kinetic barrier to fusion immediately prior to hemifusion [61]. We previously observed that the formation of protrusions during reconstitution of docking and priming is dependent on the presence of VAMP2 [48]. Most likely, interactions of Syt1 with the GUV membrane and the assembling trans‐SNARE complexes lead to an accumulation of membrane‐deforming proteins at the fusion site which would promote protrusion formation. In addition, the transmembrane domain of syntaxin 1A and the C2B domain of Syt1 both bind to PIP_2_, and local‐lipid clustering may further contribute to protrusion formation [62, 63]. In the current study, we have sufficient data quality to assess the presence of protein densities and find that protrusions are formed when proteins are clustered at the docking site, which occurs at larger (typically > 5 nm) membrane separations.

The majority of clustered docking sites are found with membrane separations between 5 and 15 nm; however, several junctions were also found at distances between 15 and 26 nm. If VAMP2 is folded to its zero layer when bound to a partially assembled t‐SNARE and the unfolded regions are extended chains with an average length of 0.365 nm per amino acid, then the partially folded SNAREpin could bridge a membrane separation of ~ 25–26 nm [39]. This is consistent with the longest GUV‐SUV membrane separations observed here of 27 nm (in the δ‐84 sample) Fig. 4A. However, due to the small persistence length of a polypeptide, it is unlikely that the partially zippered SNARE complex covers this maximum distance. The small number of very long distance contacts may represent Syt1‐PIP_2_/t‐SNARE interactions in the absence of SNAREpin formation or a minor fraction of misaligned trans‐SNARE complexes.

Half‐zippered SNAREpins have been estimated to bridge a membrane separation of ~ 10 nm [57]. In our *in vitro* system, the majority of docked sites are therefore likely to have SNAREpins zippered at least to the 0‐layer. As the membranes of the vesicle and target membrane get closer together, it is possible that further zippering has occurred, with a membrane separation between 3 and 8 nm. At this separation, protein complexes are partially excluded from between the vesicle and target membrane, and move radially outwards. This reduces the local clustering of protein densities on the GUV membrane, and protrusions are no longer seen Fig. 3C. We speculate that the protrusions observed in clustered junctions may represent an intermediate on the way to the completely primed SNAREpin.

When membranes are within 3 nm of one another, all protein complexes are excluded from the region of close approach between the membranes and are found in a ring‐like arrangement around the contact site. Ordered oligomeric rings formed by the C2B domains of Syt1 molecules varying in diameter from 18 to 43 nm (average diameter of 28 nm) have been observed in recent *in vitro* structural studies [41, 58]. The C2B domains partially insert into phospholipid membranes and interact with t‐SNARE helices via the so‐called primary interface [35, 39]. In addition, the C2B domains of other Syt1 molecules interact with the opposite side of the SNAREpin via a tripartite interface also containing CpxI [40]. In the absence of Ca^2+^, these oligomeric, ring‐like Syt1 structures would constrain the SNAREs from zippering and fusion pore opening [35]. The protein rings that we observed near the GUV membrane in ring‐like junctions varied considerably in their completeness, diameter and appearance Fig. S1. The rings may result from simple steric exclusion of fusion protein complexes from the contact site due to membrane proximity, or their formation may induce increased membrane proximity. We note that these two possibilities are not exclusive and a mixed model is possible. While our data do not allow us to determine the composition of the rings or the mechanism by which they form, seen in the context of existing literature we are tempted to speculate that they represent Syt1 rings that are less regular and more incomplete than those observed in simpler *in vitro* systems. Further experiments to disrupt Syt1 oligomerisation [58], [44], [64], or to obtain higher spatial resolution will be required to resolve the molecular organisation of the ring‐like arrangements.

The number of protein complexes at fusion junctions has also been a subject of intense debate. Recent cryo‐ET studies of vitrified synapses suggested a possible sixfold symmetrical arrangement of fusion protein complexes at primed junctions [44]. Another recent study using a reconstituted SUV system observed a variable number of protein complexes at sites of docking, while finding that sites of point‐contact contain smaller numbers of protein complexes (six or fewer) [45] to enable fast millisecond rates of fusion, consistent with coarse grain models [65]. Larger ring‐like contacts such as those seen in our data were not observed [45], perhaps reflecting the use of SUV rather than flatter GUV target membranes in the reconstitution. In our *in vitro* reconstitution, we also observed highly variable numbers of protein densities at primed prefusion junctions, and highly heterogeneous morphologies at fusion sites. In this reconstituted system, nearly all primed prefusion SUVs fuse upon calcium addition [47]. This leads us to conclude that docking and priming can be achieved with heterogeneous protein arrangements and stoichiometry and that the organisation of protein complexes at fusion junctions correlates with membrane proximity. Moreover, fusion junctions seem to have evolved great plasticity in their ability to not only dock vesicles but also fuse them with very low and very large numbers of protein complexes. This plasticity may make the system more robust. Nevertheless, *in vivo*, in particular at the neuronal synapse, with synaptic vesicles of defined diameters and very fast exocytosis, other regulators including Munc13 likely coordinate with Munc‐18 and Syt1 to more precisely define the copy number and arrangement of SNARE protein assemblies.

## Conflict of interest

JAGB is an academic editor of FEBS Letters.

## Author contributions

LG, JM, and AFPS performed experiments. LG and DM analysed images. LG, JM and AS prepared reagents. JM, THS and JAGB designed experiments. LG, JM, AFPS, THS and JAGB interpreted data. LG, JM, THS and JAGB wrote the paper with input from all authors.

## Supporting information


**Fig. S1.** Slices from subtomograms depicting junctions from the ring‐like protein distribution class.
**Fig. S2.** Total number of protein electron densities at ring‐like, intermediate and clustered fusion sites.

**Video S1.** Animation of slices through subtomogram volumes of ring‐like, intermediate and scattered fusion sites.

**Video S2.** Animation of averaged junction volumes from the ‘Clustered’, ‘Intermediate’ and ‘Ring‐like’ junctions.

## Data Availability

A representative tomogram has been deposited in the Electron Microscopy Data Bank (EMDB) under accession number EMD‐11628. All tomograms, together with the coordinates of docking sites, have been deposited in the Electron Microscopy Public Image Archive under accession number EMPIAR‐10498.
