# Supplementary material for: Arrangements of proteins at reconstituted synaptic vesicle fusion sites depend on membrane separation
Source: FEBS Lett. 2020 Sep 12;594(21):3450–63. doi: 10.1002/1873-3468.13916 (PMC7711843; doi:10.1002/1873-3468.13916)
Supplement: Supplementary file 1 — Fig. S1. Slices from subtomograms depicting junctions from the ring‐like protein distribution class. Fig. S2. Total number of protein electron densities at ring‐like, intermediate and clustered fusion sites. [file FEB2-594-3450-s001.pdf]

Supplementary Figures

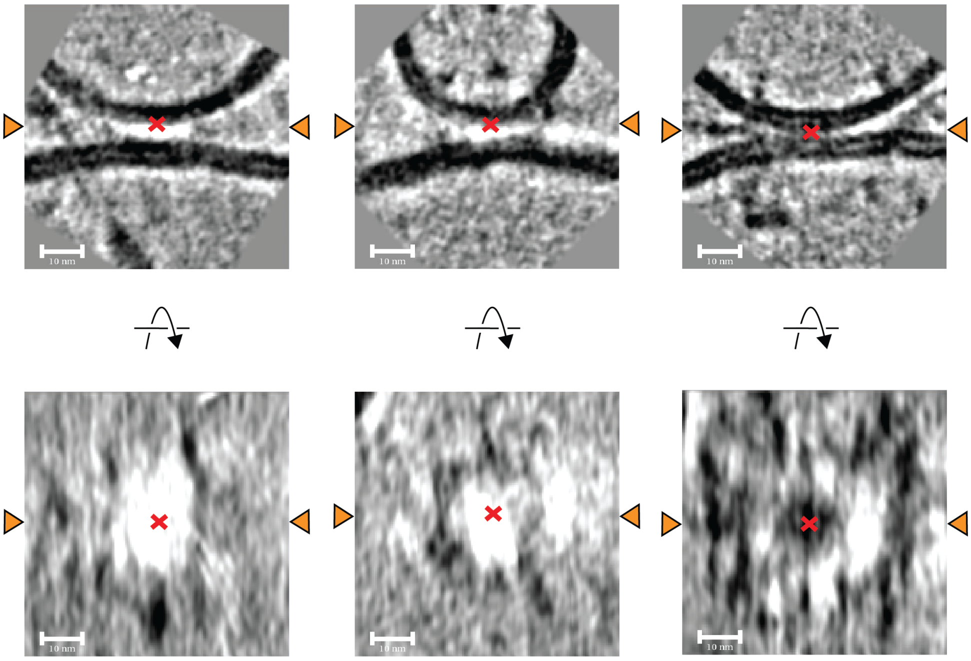

Supplementary figure 1: **Slices from subtomograms depicting junctions from the ring-like protein distribution class.** Crosses in upper images denote the base of SUVs, in lower images crosses denote the position of the SUV base projected onto slices. Orange arrowheads indicate the line at which the two slices intersect. Scalebars = 10 nm. Ring-like junctions displayed heterogeneous protein density arrangements and different degrees of 'ring completeness'.

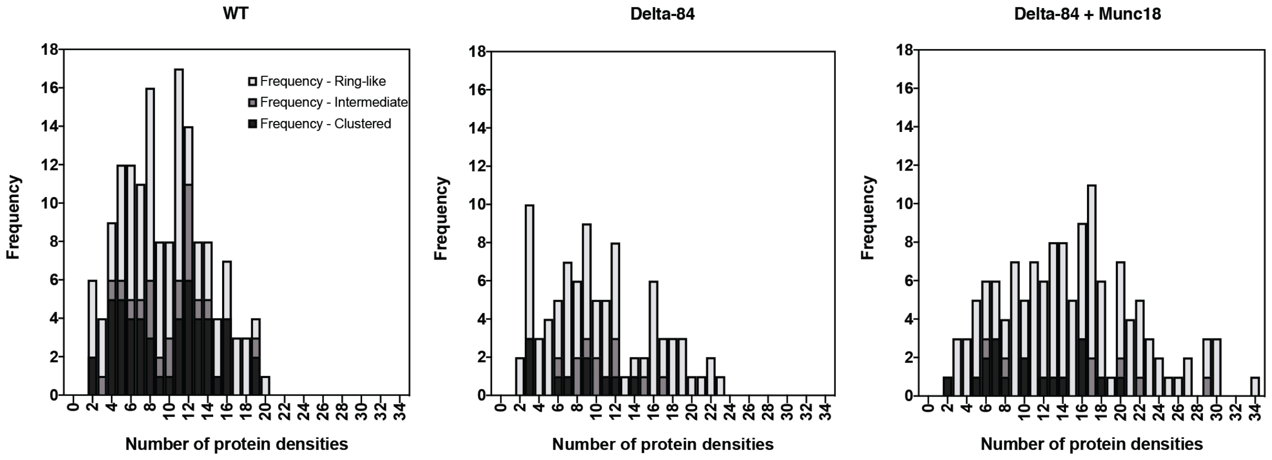

Supplementary figure 2. Total number of protein electron densities at ring-like, intermediate and clustered fusion sites. Stacked histograms displaying the frequency of protein density numbers for the WT, δ-84 and δ-84 + Munc18 conditions. Note that the number of observed protein densities will be lower than the number of proteins.

Supplementary Video Legends

Supplementary video 1. **Animation of slices through subtomogram volumes of ring-like, intermediate and scattered fusion sites.** Exactly as shown in **Figure 3**, slices are shown through representative subtomograms perpendicular to the membrane (top panel), or parallel to the membrane (bottom panel). Sequential slices through the volume, are shown, moving forwards and backwards. The bottom panels start from the GUV membrane, moving towards the SUV membrane.

Supplementary video 2. **Animation of averaged junction volumes from the 'Clustered', 'Intermediate' and 'Ring-like' junctions.** Exactly as shown in **Figure 6**, individual subtomograms of each protein distribution junction class were aligned and averaged. The coordinate points of protein densities from all junctions of that class are overlaid onto the averaged volumes as orange spheres. Volumes are rotated to allow 3D visualisation.
